# Enteral Feeding Practices for Very Preterm and Very Low Birth Weight Infants in Nigeria and Kenya

**DOI:** 10.3389/fped.2022.892209

**Published:** 2022-05-11

**Authors:** Olukemi O. Tongo, Macrine A. Olwala, Alison W. Talbert, Helen M. Nabwera, Abimbola E. Akindolire, Walter Otieno, Grace M. Nalwa, Pauline E. A. Andang'o, Martha K. Mwangome, Isa Abdulkadir, Chinyere V. Ezeaka, Beatrice N. Ezenwa, Iretiola B. Fajolu, Zainab O. Imam, Dominic D. Umoru, Ismaela Abubakar, Nicholas D. Embleton, Stephen J. Allen

**Affiliations:** ^1^College of Medicine, University of Ibadan/University College Hospital, Ibadan, Nigeria; ^2^Jaramogi Oginga Odinga Teaching and Referral Hospital, Kisumu, Kenya; ^3^KEMRI-Wellcome Trust Research Programme, Kilifi, Kenya; ^4^Liverpool School of Tropical Medicine, Liverpool, United Kingdom; ^5^Alder Hey Children's Hospital NHS Trust, Liverpool, United Kingdom; ^6^Department of Nutrition and Health, Maseno University, Maseno, Kenya; ^7^Ahmadu Bello University Teaching Hospital, Zaria, Nigeria; ^8^College of Medicine, University of Lagos/Lagos University Teaching Hospital, Lagos, Nigeria; ^9^Lagos State University Teaching Hospital, Lagos, Nigeria; ^10^Maitama District Hospital, Abuja, Nigeria; ^11^Newcastle University, Newcastle upon Tyne, United Kingdom; ^12^The Newcastle upon Tyne Hospitals NHS Foundation Trust, Newcastle upon Tyne, United Kingdom

**Keywords:** feeding practices, very preterm, very low birth weight, Nigeria, Kenya

## Abstract

**Background:**

Optimizing nutrition in very preterm (28–32 weeks gestation) and very low birth weight (VLBW; 1,000 g to <1,500 g) infants has potential to improve their survival, growth, and long-term health outcomes.

**Aim:**

To assess feeding practices in Nigeria and Kenya for very preterm and VLBW newborn infants.

**Methods:**

This was a cross-sectional study where convenience sampling was used. A standard questionnaire was sent to doctors working in neonatal units in Nigeria and Kenya.

**Results:**

Of 50 respondents, 37 (74.0%) were from Nigeria and 13 (26.0%) from Kenya. All initiated enteral feeds with breastmilk, with 24 (48.0%) initiating within 24 h. Only 28 (56.0%) used written feeding guidelines. Starting volumes ranged between 10 and 80 ml/kg/day. Median volume advancement of feeds was 20 ml/kg/day (IQR 10–20) with infants reaching full feeds in 8 days (IQR 6–12). 26 (52.0%) of the units fed the infants 2 hourly. Breastmilk fortification was practiced in 7 (14.0%) units, while folate, iron, calcium, and phosphorus were prescribed in 42 (84.0%), 36 (72.0%), 22 (44.0%), 5 (10.0%) of these units, respectively. No unit had access to donor breastmilk, and only 18 (36.0%) had storage facilities for expressed breastmilk. Twelve (24.0%) used wet nurses whilst 30 (60.0%) used formula feeds.

**Conclusion:**

Feeding practices for very preterm and VLBW infants vary widely within Nigeria and Kenya, likely because of lack of locally generated evidence. High quality research that informs the feeding of these infants in the context of limited human resources, technology, and consumables, is urgently needed.

## Introduction

Globally, about 20.5 million newborn infants were born with birthweights <2,500 g in 2015, 90% of whom were from low-and middle-income countries (LMICs) (1, 2). Nearly half of under 5 deaths are among neonates (infants <28 days old) (3). Eighty percent of neonatal deaths occur in low birthweight (LBW) infants, which includes both preterm infants born before 37 completed weeks gestational age and infants who are small for gestational age (SGA) i.e., weight <10th percentile for gestational age. Preterm birth is the single most important cause of death in the neonatal period accounting for up to a million neonatal deaths annually (2, 4, 5). Amongst LBW infants, very low birth weight (VLBW; 1,000 g to <1,500 g), and very preterm (born 28 to <32 weeks gestational age) are even more at risk, with higher incidences of late onset sepsis (LOS), necrotising enterocolitis (NEC), feeding intolerance and ultimately, mortality (6, 7).

Optimizing early nutrition in very preterm and VLBW neonates has the potential to improve their survival, growth, neurodevelopment, and long-term health outcomes. Early feeding strategies for preterm infants vary widely across the world and, although optimal postnatal growth rates have not been established, there is a general consensus to aim for a gestation-equivalent fetal growth rate (8). Noteworthy is that preterm infants have higher nutritional requirements than term infants. To achieve this, an energy intake of 110 to 135 Kcal/kg/day and protein intake of 3.5 to 4 g/kg/day in VLBW infants is recommended (9). Failure to meet recommended nutrient intakes results in poor growth and is associated with increased short-term risks such as LOS and predisposes them to long-term neurodevelopmental impairment and adult onset metabolic and cardiovascular disease (8, 10–12).

The majority of available evidence on feeding strategies in hospitalized very preterm/VLBW infants is derived from high income countries (HICs) with limited data from sub-Saharan Africa (sSA) (13). The implementation of recommended strategies is fraught with challenges in the context of resource limitations, a common problem in sSA. Early initiation of enteral feeds and exclusive feeding with breastmilk and fortification of human milk for hospitalized very preterm/VLBW babies is common in high income countries due to the availability of breastmilk banks and fortifiers, which are not available in most centers in sSA (14). In addition, early parenteral nutrition used in HICs, to provide the necessary nutrients whilst full enteral feeds are established, is not widely available and affordable in most of sSA.

In 2011, in recognition of these challenges in LMICs, the World Health Organization (WHO) emphasized early and exclusive breastmilk for preterm babies with formula supplementation only in infants with sub-optimal growth trajectories (15), by which stage key periods for brain growth and differentiation may have been missed. Evidence-based feeding guidelines require high quality research and are essential in resource constrained settings. To achieve this, it is essential to collect data on existing feeding practices.

We conducted a survey to describe feeding practices in hospitalized very preterm/VLBW infants among neonatal care practitioners in Nigeria and Kenya as part of the Neonatal Nutrition Network project (https://www.lstmed.ac.uk/nnu), to identify the diverse challenges and mitigating factors in the context of limited resources. These data will inform the prioritization and design of guidelines and interventions to optimize nutrition in these vulnerable infants in sSA.

## Methodology

### Study Design and Setting

This was a cross-sectional survey conducted between February 1, 2018, and April 30, 2019 among pediatricians and neonatologists working in neonatal units in Nigeria and Kenya. Convenience sampling was used.

### Study Population and Sampling

A standard questionnaire was sent to doctors working in neonatal units in public and private hospitals in Nigeria and Kenya through the mailing lists of the Nigerian Society of Neonatal Medicine (NISONM) (16) and the Kenya Paediatric Association (KPA) (17). Additional participants (neonatologists) were approached during a workshop on neonatal nutrition in Ibadan, Nigeria, in March 2018. The questionnaires were anonymized although respondents had the option to provide their names. Names of the hospitals and the level of care provided were requested as well as the designation of the respondents. In Nigeria, where there were multiple responses from individual participating centers, that of the most senior doctor was selected. In Kenya, individual clinicians were approached from each hospital.

### Data Collection

The questionnaire was emailed to Nigerian Society of Neonatal Medicine (NISONM) members and returned by e mail. Online forms prepared using REDCap software were emailed to members of the Kenya Paediatric Association (KPA). The questions included the number and level of personnel working in the doctor's neonatal unit and the available equipment and laboratory services. Information on the number of patients, reasons for admission and the feeding practices including time of first feed, the type of feeds, starting volumes and advancement rates as well as use of supplements were also sought.

### Statistical Analysis

Data were entered on an Excel spreadsheet which was then transferred to Stata 15 (StataCorp, College Station, Texas, USA) for statistical analysis. Summary statistics were calculated: frequencies, means with standard deviation (SD) for normally distributed data and medians with interquartile ranges (IQR) for non-parametric data.

## Results

A total of 152 questionnaires were sent out, 48 in Nigeria and 104 in Kenya. A total of 50 were returned representing 37 (74.0%) different hospitals in Nigeria and 13 (26.0%) in Kenya. Table 1 shows the distribution of the centers according to level of health care provided.

**Table 1:** Level of neonatal care where participants worked by country.

	**No of Units**	**Total**
	**Nigeria**	**Kenya**	
Secondary level	8	12	20
Tertiary level	29	1	30
Total	37	13	50

### Available Personnel and Services Provided

Neonatal unit size ranged from 2 to 58 cots/incubators; median capacity was 22 (IQR 11–32). All but 2 of the hospitals had neonatologists or pediatricians. The median number of combined neonatologists/pediatricians attending each unit was 9.0 (IQR 3.5–18.0); the median was 10.0 (IQR 4.0–20.0) in Nigeria and 5.0 (IQR 2.5–8.5) in Kenya. Table 2 shows the level of care, equipment, and services available in the neonatal units across both countries. There were few hospitals with functioning equipment for respiratory support: CPAP machines (22%) and ventilators (8%). Only 31/50 (62%) hospitals reported availability of amino acid preparations for parenteral nutrition. Kangaroo mother care was used in all the Kenyan hospitals in the survey and in 86% of the Nigerian hospitals.

**Table 2:** Level of care, investigations, equipment, and services available in the neonatal units.

	**Nigeria (*N =* 37) *n* (%)**	**Kenya (*N =* 13) *n* (%)**	**Both countries (*N =* 50) *n* (%)**
**Equipment/consumables Available**
Functioning ventilator(s)	2 (5.4)	2 (15.4)	4 (8.0)
Functioning Continuous Positive Airway Pressure (CPAP) machine(s)	3 (8.1)	8 (61.5)	11 (22.0)
Appropriately sized intravenous cannulas	27 (73.0)	9 (69.2)	36 (72.0)
Peripheral long lines	17 (45.9)	1 (7.7)	18 (36.0)
Umbilical venous catheters	26 (70.3)	4 (30.8)	30 (60.0)
Umbilical artery catheters	8 (21.6)	0 (0.0)	8 (16.0)
Supplemental parenteral nutrition (amino- acids only)	25 (67.6)	6 (46.2)	31 (62.0)
**Investigative Capacity**
Microbiology laboratories	34 (91.9)	10 (76.9)	44 (88.0)
X-ray machines	35 (94.6)	13 (100.0)	48 (96.0)
Ultrasonography	33 (89.2)	9 (69.2)	42 (84.0)
**Other Services**
Kangaroo Mother Care (KMC)	32 (86.5)	13 (100.0)	45 (90.0)

*n, number of facilities*.

### Spectrum of Neonates Treated in the Units

**Figure 1:**
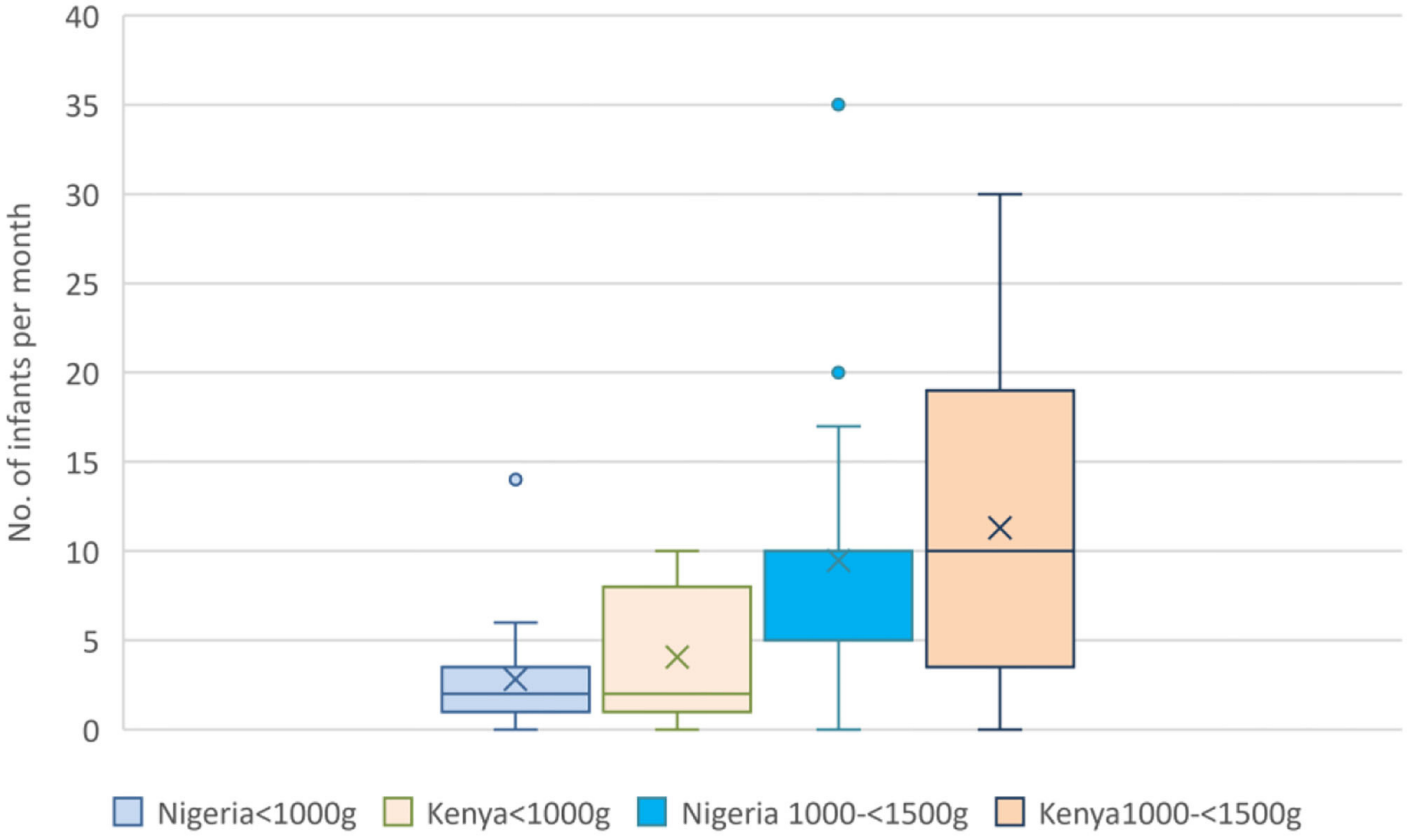
Reported number of admissions of very low birthweight infants per month. Boxes show the IQR with solid line marking the median; X marks the mean; whiskers mark “minimum” (1st quartile-1.5 IQR) and “maximum” (3rd quartile +1.5 IQR) values; dots show outliers.

Thirty-nine (78.0%) units accepted babies born at home (outborn) for admission into the same ward and inborns; the remainder admitted outborns to a separate area such as the general pediatric ward. Figure 1 shows the reported number of babies admitted per month according to birthweight category. Babies with birthweight <1,500 g constituted around a third of all neonatal admissions (median 33.3%; IQR 20–44%). The median number of infants with birthweight <1,500 g admitted per center per month in both countries was 12.0 (IQR 4.8–18.0). Forty-seven centers (94.0%) used postnatal clinical scoring systems such as Dubowitz and Ballard for gestational age assessment. There were no responses on the proportion of mothers with access to early (first trimester) ultrasound scans in pregnancy from the Kenyan units; in Nigeria, 40% were reported to have had access to early ultrasound scans.

### Feeding Practices and Clinical Guidelines for Very Preterm and VLBW Babies

Written feeding guidelines for very preterm/VLBW infants were available in 17 (45.9%) of the Nigerian units and 11 (84.6%) of the Kenyan units. All respondents reported initiating enteral feeds with expressed breastmilk. Feeding practices are shown in Table 3 below. The median volume of advancement of feeds was 20 ml/kg/day (IQR 10–20). The median time to full enteral feeds (defined in the questionnaire as 120 ml/kg/day) was 8 days (IQR 6–12) and the range was from 3 to 20 days. Routine assessment of gastric residual volume before tube feeding was practiced in 39/50 (78.0%) centers. 36 (72.0%) respondents reported that enteral feeds are withheld from babies at key times depending on gestational age, asphyxia, or severe intrauterine growth restriction.

**Table 3:** Reported feeding practices.

	**Nigeria (*N =* 37) *n* (%)**	**Kenya (*N =* 13) *n* (%)**	**Both countries (*N =* 50) *n* (%)**
**Time to first feed**
Within first 24 h of life	16 (43.2)	8 (61.5)	24 (48.0)
>24 to 48 h of life	12 (32.4)	3 (23.1)	15 (30.0)
>48 h to 72 h of life	7 (18.9)	2 (15.4)	9 (18.0)
>72 h of life	2 (5.4)	0 (0.0)	2 (4.0)
**Starting volume of feeds**
10–20 ml/kg/day	34 (91.9)	2 (15.4)	36 (72.0)
40–80 ml/kg/day	3 (8.1)	11 (84.6)	14 (28.0)
**Volume of advancement of feeds**
Less than 10 ml/kg/day	1 (2.7)	1 (7.7)	2 (4.0)
10–20 ml/kg/day	31 (83.8)	11 (84.6)	42 (84.0)
More than 20 ml/kg/day	4 (10.8)	1 (7.7)	5 (10.0)
**Frequency of feeds**
Continuous	4 (10.8)	0 (0.0)	4 (8.0)
1 hourly	2 (5.4)	0 (0.0)	2 (4.0)
2 hourly	26 (70.3)	0 (0.0)	26 (52.0)
3 hourly	12 (32.4)	13 (100.0)	25 (50.0)
4 hourly	4 (10.8)	0 (0.0)	4 (8.0)
**Mode of feeding**
Nasogastric tube only	20 (54.1)	8 (61.5)	28 (56.0)
Orogastric tube only	7 (18.9)	0 (0.0)	7 (14.0)
Nasogastric and orogastric tube	8 (21.6)	3 (23.1)	11 (22.0)
Cup	26 (70.3)	10 (76.9)	36 (72.0)
Cup and spoon	11 (29.7)	4 (30.8)	15 (30.0)
Bottle	2(5.4)	1 (7.7)	3 (6.0)

### Support for Enteral Feeds

Only 3 (6.0%) practiced buccal colostrum when babies were not yet feeding by mouth and one center in Nigeria used probiotics in VLBW infants. Fortification of breastmilk was practiced in 7 (14.0%) of the units (4 in Nigeria and 3 in Kenya); none of the units had access to donor breastmilk but 12 of the 37 (32.4%) units in Nigeria engaged wet nurses when there was a shortfall in maternal breastmilk supply. None of the Kenyan units reported wet nursing. The majority of respondents, (30; 60.0%) used formula for top-up feeds. Only 18 (36.0%) of the units had storage facilities for expressed breast milk.

Nutritional supplements given were folic acid in 42 (84.0%) units, iron in 36 (72.0%), calcium in 22 (44.0%) and phosphorus in 5 (10.0%). Vitamin supplements were more often reported in units in Kenya (100.0%) than Nigeria (70.0%).

## Discussion

This survey of enteral feeding practices for very preterm and/or VLBW babies at secondary and tertiary levels of health care in Nigeria and Kenya shows very wide variations in practice within and between both countries. Although all of the units initiated feeds using expressed breast milk, only about half initiate feeding within the first 24 h and some not until after 72 h. This marked variability in practice likely accounts for the equally marked variation between units in time to reach full feeds.

Most units routinely checked gastric residual volume before oral and/or nasal tube feeding. Other modes of feeding utilized were cup, cup and spoon, and bottle. None of the units had access to donor breast milk. Formula feeds, breast milk fortifiers and wet nurses (in Nigeria only) were used to supplement shortfalls in expressed breast milk. In addition, probiotics, and nutritional supplements (folic acid, iron, calcium, and phosphorus) were widely administered. Few of the units used buccal colostrum.

Written feeding guidelines were in use in 45.9 and 85.6% of the Nigerian and Kenyan units, respectively. The use of standardized feeding protocols in middle and high income countries (18–20), is associated with earlier achievement of full enteral feeds (thus shorter use of vascular catheters), and reduced rates of neonatal sepsis, NEC, extrauterine growth restriction, and overall, decreased length of stay in the hospital. However, variations in feeding practices across units also occur in high income countries depending on a number of factors including access to facilities such as breastmilk banks (21, 22). Currently there is little research on preterm feeding practices in sSA to inform feeding protocols for these at-risk infants (13). Kenya has a national guideline for feeding these vulnerable infants (23). In Nigeria, guidelines for comprehensive newborn care in secondary and tertiary hospitals were launched on 25th November, 2021 (24). This occurred after this survey was done, thus less than half of the centers in Nigeria had unit protocols for preterm feeding. Though this study did not evaluate the degree to which facilities adhered to their protocols, it has highlighted strengths and opportunities to build on as well weaknesses and threats or challenges to address in order to successfully implement a national guideline or protocol. The implementation of national guidelines would present an opportunity for evaluation and comparison of preterm feeding across a large number of neonatal units.

The overall aim of feeding guidelines/protocols for these at-risk infants is to achieve full enteral feeds in the shortest possible time and safely, to promote immediate and long-term health. An overview of systematic reviews of feeding practices for VLBW infants in sSA (13) showed research gaps related to optimal time to starting feeds, what to feed, what volume to start with, how to advance, best mode of feeding and what supplements to use. All these practices must take into account what facilities and support are available and sustainable particularly in systems where care is paid for out of pocket.

Few units practiced administration of buccal or oropharyngeal colostrum despite this being a low-cost procedure that is being increasingly adopted in high income settings with the potential to reduce time to full enteral feeds (25). This may reflect the length of time needed for research findings to be incorporated into clinical guidelines and routine practice, the first publication dating from 2009 (26). Most units started enteral feeding with trophic feeds ranging between 10 and 20 ml/kg/day, with daily advancements of 10–20 ml/kg/day, though there is evidence from developed countries that faster advancement of 30–40 ml/kg/day may be safe and facilitates earlier attainment of full enteral feeds (8, 27) few of the units in this survey advanced more than 20 ml/kg/day. The WHO feeding guidelines for preterm infants recommend a daily increase “up to” 30 ml/kg for LMICs (14) and this may be the reason for the observed practice. The fact that the clinical status of the infants in sSA and the level of monitoring and nursing care might not be similar to those in high income countries, coupled with the suboptimal nutritional, economic and overall conditions of mothers as well as the health system set up (28) may all intricately interact to interfere with lactation, milk expression and storage and feeding regimens in these units. The WHO recommendation needs to be evaluated for infants in this region.

Multicentre studies in Africa need to factor in the particular challenges with resources such as donor breastmilk banks, with evaluation of different implementation models such as in South Africa (23). The first human breastmilk bank in Kenya commenced in Nairobi in 2019 with a view to scaling up. This process took 3 years from planning to eventual inauguration (29). No donor milk bank exists in Nigeria which may be related to resource constraints and/or cultural and religious factors. Some centers, however, utilized wet nursing to provide breastmilk. It will be informative to conduct research into the cultural acceptability and extent of this practice as well as safety particularly in the context of novel and evolving infectious diseases, as this may prove a useful and affordable alternative to breastmilk banking.

### Limitations

This study had a number of limitations. Convenience sampling was used, and the number of respondents was low, particularly from Kenya. Therefore, the findings may not be entirely representative of feeding practices in neonatal units in Nigeria and Kenya. In addition, feeding practices were self-reported and not verified from hospital records. Another limitation lies in the fact that the view of the most senior doctor in each unit was used hence it may reflect more of the intentions rather than the actual practice of junior doctors, nurses, and nutritionists involved in day-to-day decisions on feeding practices, especially in centers where there are no written feeding guidelines. Information from nurses and parents was not collected. Despite these limitations, the survey provides data from two different sSA countries to generate key context-relevant research questions.

## Conclusion

Feeding practices in very preterm/VLBW infants vary widely in Nigeria and Kenya possibly due to a complete lack of locally generated evidence to guide practice. High quality research into feeding of very preterm/VLBW infants, that is sensitive to the context of limited human resources, technology, and consumables, is urgently needed to inform the development of guidelines appropriate to these settings.

## Neonatal Nutrition Network Members

Isa Abdulkadir (Ahmadu Bello University, Zaria, Nigeria); Ismaela Abubakar (Liverpool School of Tropical Medicine, Liverpool, UK); Abimbola E. Akindolire (College of Medicine, University of Ibadan, Nigeria); Olusegun Akinyinka (College of Medicine, University of Ibadan, Nigeria); Stephen J. Allen (Liverpool School of Tropical Medicine, Liverpool, UK); Pauline E. A. Andang'o (Maseno University, Kenya); Graham Devereux (Liverpool School of Tropical Medicine, Liverpool, UK); Chinyere Ezeaka (College of Medicine, University of Lagos/Lagos University Teaching Hospital, Nigeria); Beatrice N. Ezenwa (College of Medicine, University of Lagos/ Lagos University Teaching Hospital, Nigeria); Iretiola B. Fajolu (College of Medicine, University of Lagos/ Lagos University Teaching Hospital, Nigeria); Zainab O. Imam (Lagos State University Teaching Hospital, Lagos, Nigeria); Kevin Mortimer (Liverpool School of Tropical Medicine, Liverpool, UK); Martha K. Mwangome (KEMRI Wellcome Trust Research Programme, Kilifi, Kenya); Helen M. Nabwera (Liverpool School of Tropical Medicine, Liverpool, UK); Grace M. Nalwa (Jaramogi Oginga Odinga Teaching and Referral Hospital, Kisumu, Kenya & Maseno University, Kenya); Walter Otieno (Jaramogi Oginga Odinga Teaching and Referral Hospital, Kisumu, Kenya & Maseno University, Kenya); Macrine A. Olwala (Jaramogi Oginga Odinga Teaching and Referral Hospital, Kisumu, Kenya); Alison W. Talbert (KEMRI Wellcome Trust Research Programme, Kilifi, Kenya); Nicholas D. Embleton (Newcastle University, Newcastle, UK); Olukemi O. Tongo (College of Medicine, University of Ibadan, Nigeria); Dominic D. Umoru (Maitama District Hospital, Abuja, Nigeria); Janneke van de Wijgert (University of Liverpool, Liverpool, UK); Melissa Gladstone (University of Liverpool, Liverpool, UK).

## Data Availability Statement

The raw data supporting the conclusions of this article will be made available by the authors, without undue reservation.

## Author Contributions

OT contributed to conception and design of the study and the first draft of the manuscript. MO and AT were co-authors. All authors contributed to manuscript revision, read, and approved the submitted version.

## Funding

This work was supported by an MRC Confidence in Global Nutrition and Health Research Initiative grant Improving the survival, growth and development of low birthweight newborns through better nutrition MC_PC_MR/R-19789/1.

## Conflict of Interest

The authors declare that the research was conducted in the absence of any commercial or financial relationships that could be construed as a potential conflict of interest.

## Publisher's Note

All claims expressed in this article are solely those of the authors and do not necessarily represent those of their affiliated organizations, or those of the publisher, the editors and the reviewers. Any product that may be evaluated in this article, or claim that may be made by its manufacturer, is not guaranteed or endorsed by the publisher.

## References

[1] Gu H Wang L Liu L Luo X Wang J Hou F. A gradient relationship between low birth weight and IQ: a meta-analysis. Sci Rep. (2017) 7:18035. 10.1038/s41598-017-18234-9 29269836 PMC5740123

[2] Blencowe H Cousens S Chou D Oestergaard M Say L Moller AB. Born Too Soon: the global epidemiology of 15 million preterm births. Reprod Health. (2013) 10:1–14. 10.1186/1742-4755-10-S1-S2 24625129 PMC3828585

[3] Paulson KR Kamath AM Alam T Bienhoff K Abady GG Abbas J. Global, regional, and national progress towards Sustainable Development Goal 3.2 for neonatal and child health: all-cause and cause-specific mortality findings from the Global Burden of Disease Study 2019. Lancet. (2021) 398:P870–905. 10.1016/S0140-6736(21)01207-1 34416195 PMC8429803

[4] Liu L Oza S Hogan D Chu Y Perin J Zhu J. Global, regional, and national causes of under-5 mortality in 2000–15: an updated systematic analysis with implications for the Sustainable Development Goals. Lancet. (2016) 388:3027–35. 10.1016/S0140-6736(16)31593-8 27839855 PMC5161777

[5] Blencowe H Krasevec J de Onis M Black RE An X Stevens GA. National, regional, and worldwide estimates of low birthweight in 2015 with trends from 2000: a systematic analysis. Lancet Global Health. (2019) 7:e849–60. 10.1016/S2214-109X(18)30565-5 31103470 PMC6560046

[6] Athalye-Jape G Patole S. Probiotics for preterm infants – time to end all controversies. Microbial Biotechnol. (2019) 12:249. 10.1111/1751-7915.13357 30637944 PMC6389843

[7] Nabwera HM Wang D Tongo OO Andang'o PE Abdulkadir I Ezeaka CV. Burden of disease and risk factors for mortality amongst hospitalized newborns in Nigeria and Kenya. PLoS ONE. (2021) 16:e0244109. 10.1371/journal.pone.0244109 33444346 PMC7808658

[8] Abiramalatha T Thomas N Thanigainathan S. High versus standard volume enteral feeds to promote growth in preterm or low birth weight infants. Cochrane Database Syst Rev. (2021) 3:CD012413. 10.1002/14651858.CD012413.pub3 33733486 PMC8092452

[9] Agostoni C Buonocore G Carnielli VP De Curtis M Darmaun D Decsi T. Enteral nutrient supply for preterm infants: commentary from the European Society of Paediatric Gastroenterology, Hepatology and Nutrition Committee on Nutrition. J Pediatr Gastroenterol Nutr. (2010) 50:85–91. 10.1097/MPG.0b013e3181adaee0 19881390

[10] Reyes L Manalich R. Long-term consequences of low birth weight. Kidney Int. (2005) 68:S107–11. 10.1111/j.1523-1755.2005.09718.x 16014086

[11] Mallick A. Prevalence of low birth weight in India and its determinants: insights from the national family health survey (nfhs), 2015–2016. Anthropologischer Anzeiger. (2021) 78:163–75. 10.1127/anthranz/2021/1317 33432318

[12] Crump C Sundquist J Sundquist K. Risk of hypertension into adulthood in persons born prematurely: a national cohort study. Eur Heart J. (2020) 41:1542–50. 10.1093/eurheartj/ehz904 31872206 PMC8453271

[13] Akindolire A Talbert A Sinha I Embleton N Allen S. Evidence that informs feeding practices in very low birthweight and very preterm infants in sub-Saharan Africa: an overview of systematic reviews. BMJ Paediatr Open. (2020) 4:e000724. 10.1136/bmjpo-2020-000724 32821859 PMC7422638

[14] Abhulimhen-Iyoha B Okonkwo I Ideh R Okolo A. Mothers' perception of the use of banked human milk for feeding of the infants. Nigerian J Paediatr. (2015) 42:223–7. 10.4314/njp.v42i3.10

[15] WHO. Feeding of Low-Birth-Weight Infants in Low- Middle-Income Countries. WHO. (2018). Available online at: http://www.who.int/elena/titles/full_recommendations/feeding_lbw/en/ (accessed August 19, 2021).

[16] Nigerian Society of Neonatal Medicine. Available online at: https://www.nisonm.org/ (accessed October 15, 2021).

[17] KPA. Kenya Paediatric Association. Available online at: https://kenyapaediatric.org/ (accessed October 15, 2021).

[18] McCallie KR Lee HC Mayer O Cohen RS Hintz SR Rhine WD. Improved outcomes with a standardized feeding protocol for very low birth weight infants. J Perinatol. (2011) 31:S61. 10.1038/jp.2010.185 21448207

[19] Arora S Yadav P Bajaj H Thakur AS Mittal M Gupta MR. Improving clinical outcomes of very low birth weight infants: implementation of standardized management guidelines in tertiary care hospital in Haryana. Int J Pediatr Adolesc Med. (2020) 7:174–80. 10.1016/j.ijpam.2019.08.002 33319015 PMC7729219

[20] Dutta S Singh B Chessell L Wilson J Janes M McDonald K. Guidelines for feeding very low birth weight infants. Nutrients. (2015) 7:423–42. 10.3390/nu7010423 25580815 PMC4303848

[21] Waard M de Li Y Zhu Y Ayede AI Berrington J Bloomfield FH. Time to Full Enteral Feeding for Very Low-Birth-Weight Infants Varies Markedly Among hospitals worldwide but may not be associated with incidence of necrotizing enterocolitis: the NEOMUNE-NeoNutriNet cohort study. J Parenteral Enteral Nutr. (2019) 43:658–67. 10.1002/jpen.1466 30465333 PMC6531355

[22] Berti E Puglia M Perugi S Gagliardi L Bosi C Ingargiola A. Feeding practices in very preterm and very low birth weight infants in an area where a network of human milk banks is in place. Front Pediatr. (2018) 6:387. 10.3389/fped.2018.00387 30574473 PMC6291747

[23] Ministry of Health Republic of Kenya 4th Edition (2016). Available online at: http://guidelines.health.go.ke:8000/media/Basic_Paediatric_Protocols_2016.pdf(accessed October 19, 2021).

[24] World Prematurity Day Commemoration-Nigeria Sets Additional Standards for Newborn Care. WHO | Regional Office for Africa. Available online at: https://www.afro.who.int/news/world-prematurity-day-commemoration-nigeria-sets-additional-standards-newborn-care (accessed January 5, 2022).

[25] da Cruz Martins C de Santana Xavier Ramos M Viana Cardoso Amaral M. Colostrum oropharyngeal immunotherapy for very low birth weight preterm infants: protocol of an intervention study. BMC Pediatr. (2020) 20:1–11. 10.1186/s12887-020-02266-8 32767992 PMC7411269

[26] Rodriguez NA Meier PP Groer MW Zeller JM. Oropharyngeal administration of colostrum to extremely low birth weight infants: theoretical perspectives. J Perinatol. (2009) 29:1. 10.1038/jp.2008.130 18769379 PMC2730520

[27] Raban S Santhakumaran S Keraan Q Joolay Y Uthaya S Horn A. A randomised controlled trial of high vs low volume initiation and rapid vs slow advancement of milk feeds in infants with birthweights ≤ 1000 g in a resource-limited setting. Paediatr Int Child Health. (2016) 36:288–95. 10.1179/2046905515Y.0000000056 26369284

[28] Kruk ME Gage AD Arsenault C Jordan K Leslie HH Roder-DeWan S. High-quality health systems in the Sustainable Development Goals era: time for a revolution. Lancet Global Health. (2018) 6:e1196–252. 10.1016/S2214-109X(18)30386-3 30196093 PMC7734391

[29] CICF Learning Series Evidence Brief,. Helping Babies Thrive: Establishing the First Integrated Human Milk Bank System in Kenya 2019 (2019). Available online at: https://www.options.co.uk/sites/default/helping_babies_thrive_hmb.pdf(accessed August 19, 2021).

